# Comparing the Effects of Different Unsaturated Fatty Acids on Fermentation Performance of *Saccharomyces cerevisiae* and Aroma Compounds during Red Wine Fermentation

**DOI:** 10.3390/molecules24030538

**Published:** 2019-02-01

**Authors:** Pei-Tong Liu, Chang-Qing Duan, Guo-Liang Yan

**Affiliations:** 1 Centre for Viticulture and Enology, College of Food Science and Nutritional Engineering, China Agricultural University, Beijing 100083, China; pt_liu@cau.edu.cn (P.-T.L.); duanchq@vip.sina.com (C.-Q.D.); 2 Key Laboratory of Viticulture and Enology, Ministry of Agriculture, Beijing 100083, China

**Keywords:** linoleic acid, oleic acid, α-linolenic acid, *Saccharomyces cerevisiae*, volatile aroma compounds, red wine

## Abstract

To understand the individual enological function of different unsaturated fatty acids (UFAs), the separated effects of three different UFAs, linoleic acid (LA), oleic acid (OA), and α-linolenic acid (ALA), on yeast fermentation and aroma compounds were investigated in the alcoholic fermentation of Cabernet Sauvignon wine. The results showed that, besides concentration, UFAs types could also influence fermentation process and volatiles in final wine. Low concentrations of UFAs (12 and 60 mg/L), especially LA and OA, significantly promoted fermentation activity and most volatiles when compared to the control, however, the effect became the inhibition with increasing concentrations of UFAs (120 and 240 mg/L). It was interesting to find that OA addition (12 and 60 mg/L) could generate more acetate esters (especially isoamyl acetate) in wine, while 12 mg/L LA facilitated more fatty acids formation (octanoic acid and decanoic acid). In comparison, 120 and 240 mg/L ALA produced more amount of C6 alcohols (1-hexanol) and higher alcohols (isobutyl alcohol and 2,3-butanediol). UFAs additions were unfavorable for ethyl esters formation, except for an increment of ethyl hexanoate in 12 mg/L OA wine. As a result, different aromatic profiles of wines were generated by variations of UFAs types and levels, as shown by PCA. The transcriptional data revealed that the expressions of aroma-related genes, such as *BAT1*, *BAT2*, *PDC1*, *PDC5*, *PDC6*, *ACC1*, *FAS1*, *ATF1*, *EEB1,* and *EHT1* were correlated with aroma compounds productions in different treatments. Our data suggested that the three UFAs have different enological functions and they could generate different aromatic profiles. Thus, besides concentrations, it is essential to consider the types of UFAs when applying the strategy to adjust UFAs contents to modulate the aromatic quality of wines.

## 1. Introduction

Grape must is a very complex medium, with at least 200 g/L of sugars and hundreds of other grape metabolites, including amino and non-amino organic acids and fatty acids with concentrations varying from ng/L to g/L [1,2]. It is well-known that the composition of musts has a vital role in determining the aroma profile that is produced by the fermenting yeast. Even small changes in the must composition could result in a critical effect on the growth and metabolism of wine yeasts, and thus alter the formation of aroma compounds [3]. Numerous studies have been conducted to determine the role of major compounds (such as sugar and amino acid) during wine fermentation [2,4,5], however, little information is available on unsaturated fatty acids (UFAs). In fact, as the essential nutrients for yeast to grow under anaerobic conditions, UFAs are crucial for yeast to maintain membrane integrity, as well as the function to adapt well to fermentation stresses [6]. More importantly, UFAs can directly affect the formation of volatile compounds (including medium-chain fatty acids (MCFAs) and esters) by regulating the synthesis of precursor acyl-CoA and the expression of related genes [7,8,9].

Grape berries contain 0.15% to 0.24% (wet weight basis) lipids [10]. UFAs are the major components of the total lipids in grape berries, in which linoleic acid (C18:2, LA) is the most abundant, followed by oleic acid (C18:1, OA) and α-linolenic acid (C18:3, ALA). Their content varied from 0.04 mg/L to 280 mg/L in grape must, and changed with the grape cultivars [11,12], fermentation technologies, such as grape must clarification and grape-skin maceration [13]. In comparison, the fatty acids of *Saccharomyces cerevisiae* are mainly made up of OA and palmitoleic acid (C16:1), together with the saturated fatty acids (SFAs), palmitic acid (C16:0), and stearic acid (C18:0) [9]. Due to lacking Δ12-fatty acid desaturase and ω3-fatty acid desaturase, *S. cerevisiae* cannot produce polyunsaturated fatty acids (PUFAs), such as LA and ALA [9]. However, during wine fermentation, the absence of oxygen suppresses fatty acid desaturation of yeast. An alternative to the biosynthesis is the direct uptake of UFAs from the grape juice. The incorporate exogenous fatty acids into cells can significantly modify the lipid composition of the cells and influence the physiological status and metabolism of yeast [9,14,15,16]. Several studies had investigated the effect of exogenous UFAs on wine aroma biosynthesis; however, the results were not consistent. For example, the addition of OA (31 mg/L) and ergosterol (25 mg/L) increased higher alcohols and acetate esters production, except 1-butanol and 1-pentanol, which exhibited an opposite trend [17]. While Thurston et al. found that the presence of LA (50 mg/L) can suppress the ethyl esters and acetate esters production [18]. Recently, Casu et al. reported that increasing the concentration of LA (132 mg/L) was unfavorable for acetate esters formation, but improved the productions of higher alcohols [14]. In one study of a synergistic effect, Tween 80, containing 70% of OA and 30% of palmic acid and stearic acid, was added and improved the esters, higher alcohols and volatile fatty acids of wine [19]. Our previous work indicated that the rational controlling of UFAs contents can improve volatile compounds in synthetic grape medium, such as 2-phenylethanol, butanoic acid, hexanoic acid, isoamyl acetate, and 2-phenylethyl acetate [15]. These inconsistent results could be ascribed to the differences of added UFAs concentration, medium composition, yeast strain and fermentation conditions, while the type of UFAs should not be overlooked because the physiological functions of different UFAs are also different. For example, the ability of PUFAs (LA and ALA) to maintain membrane fluidity is higher when compared to monounsaturated fatty acids (MUFAs, such as OA) and thus showed better protection to cells in harsh conditions [15]. This might simultaneously lead to the variation of activity of ATPase and general amino acid permease because the higher fluidity of cell membrane is essential for guaranteeing the activities of membrane-associated enzymes during stressed conditions [6,20]. The physiological differences of UFAs might result in a different metabolic response of yeast to the variation of UFAs types during wine fermentation, and lead to different aromatic profiles of the final wine. To our knowledge, no related works have been reported so far. To broaden the understanding of types and concentrations of UFAs influence on the aroma profile of wine, and try to reveal the mechanism of UFAs different effects, in this work, the separated effect of OA, LA, and ALA on cell growth, fermentation kinetics, and aroma compounds were investigated. To achieve this purpose, different levels of OA, LA, and ALA were initially supplemented into Cabernet Sauvignon juice, respectively, and fermented by wine yeast S. cerevisiae EC1118. To reveal the mechanism, the transcriptional profiles of aroma-related genes during fermentation were further determined.

## 2. Results and Discussion

### 2.1. Yeast Growth and Fermentation Profiles

Yeast cell growth (DCW g/L) and sugar consumption in different treatments were monitored during fermentation (Figure S1). To facilitate comparison, the key kinetic parameters, including the duration of fermentation, maximum biomass, the time to reach maximum biomass, maximum specific growth rate, and fermentation rate were calculated and are shown in Table 1. Except for OA_240, LA_240, ALA_120, and ALA_240, which last for 444 h of fermentation, other treatments successfully completed the alcoholic fermentation (residual sugar content below 2.5 g/L) (Figure S1). This suggested that high concentration of OA, LA, or ALA in initial grape must could result in slow alcoholic fermentation, especially in ALA wine. In comparison, low added concentrations (12 and 60 mg/L) favored yeast growth, and consequently resulted in improved fermentation activity, especially in LA and OA wines. For example, 12 mg/L LA addition shortened 154 h and 36 h of fermentation time compared to the control and ALA_12 wine, respectively, and the highest maximum biomass (3.08 g DCW/L) and fermentation rate (0.023/h) were correspondingly achieved in LA_12 wine. These results were consistent with the results of Redón et al. [21], whereby exogenous UFAs are favorable for cell growth and fermentation activity. However, our data indicated that the effects of UFAs on cell growth and fermentation performance are largely dependent on the concentration and type of UFAs. Promotion only happened in low concentrations (12 and 60 mg/L), in which LA showed the highest promotion, followed by OA and ALA. Conversely, high addition could result in the inhibition, especially ALA.

**Figure 1:**
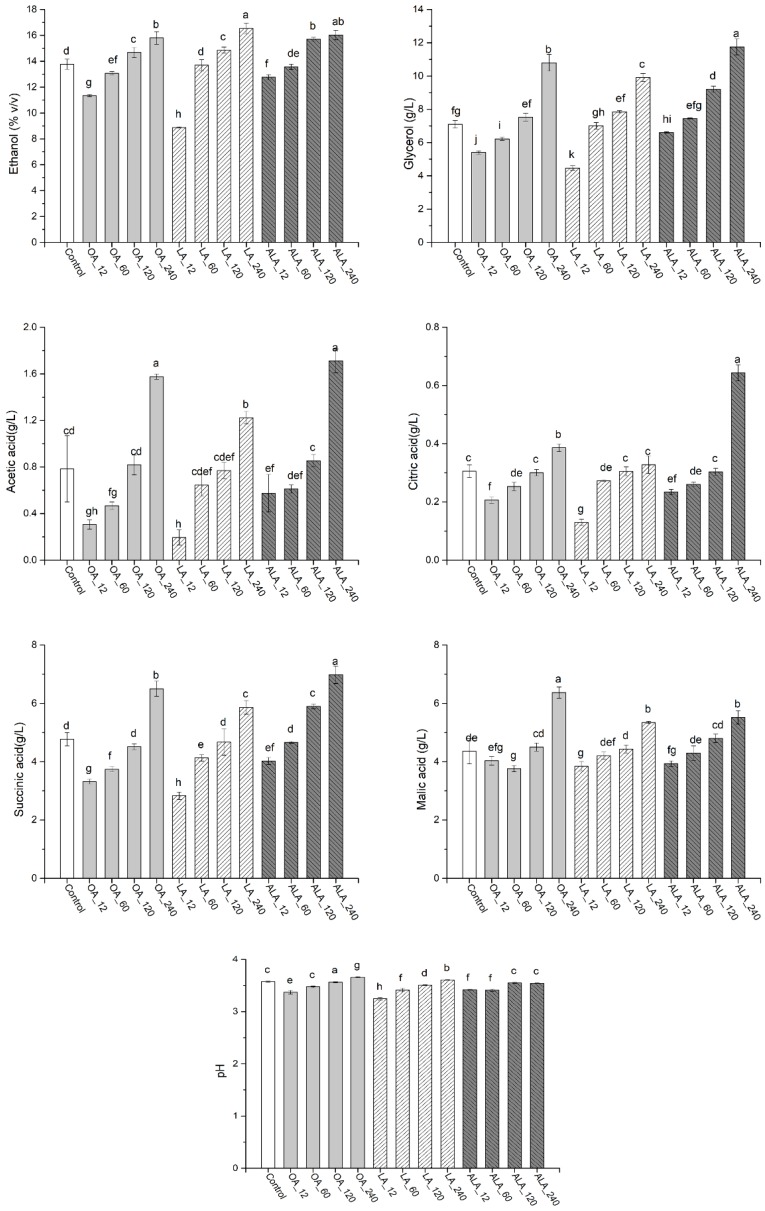
Effect of different treatments on the composition of wines. Values of the same compound marked with the same lowercase letters indicates no significant difference (*p* < 0.05, Duncan’s test).

The physiochemical parameters of final wines were determined, including ethanol, glycerol, citric acid, malic acid, succinic acid, and acetic acid (Figure 1). The variations of UFAs concentration and type significantly influenced the production of these metabolites. The additions of 12 and 60 mg/L UFAs, especially 12 mg/L, reduced the contents of ethanol, glycerol, acetic acid, citric acid, and succinic acid, in which LA_12 had a pronounced effect, followed by OA_12. Excessive ethanol can mask the flavor profile of wines and negatively influence consumer’s health, which increases the demand for reducing alcohol concentration in wines [3]. Our results suggested that slightly increasing LA and OA concentration in grape must can decrease ethanol formations, which might be a potential strategy to reduce ethanol content in wine. High contents of acetic acid and succinic acid can generate vinegar-like character and bitter taste to wine [8]. 12 and 60 mg/L UFAs addition decreased the productions of acetic acid and succinic acid. What is more, the pH of these treatments was also lower than others. It should be noticed that further increasing UFAs concentration resulted in promotion on most of the metabolites, especially ALA and OA additions. The contents of acetic acid, glycerol, citric acid, and succinic acid in ALA_240 were increased by 2.18-, 1.65-, 2.11-, and 1.46-fold when compared to the control, which were also 1.40-, 1.19-, 1.96-, and 1.19-fold higher than those of LA_240 wine, respectively. The significant influence that is caused by UFAs in this study was not consistent with Duan et al., who found no pronounced difference among UFAs treatments and control [15]. This might be due to the different type and concentration of UFAs, as well as the different fermentation mediums (grape must in this study and synthetic medium in Duan’s work).

We further determined the fatty acids composition of cells that were treated by different UFAs after alcoholic fermentation, including saturated fatty acids (SFAs), UFAs contents, and the ratio of UFAs/SFAs (Figure S2). Except for LA_120 and LA_240 samples, UFAs addition decreased the total contents of SFA (octadecanoic acid and hexadecanoic acid), especially in ALA_120 and ALA_240 samples, and the decrement were mainly caused by the reduction of hexadecanoic acid. Extracellular UFAs can be incorporated into yeast cells and increase the concentration of the corresponding UFA in cell membrane [15,22]. Among three UFAs, LA showed the highest promotion, as the highest content of UFAs was observed in the LA_240 sample, which was 6.1-, 1.9-, and 4.0-fold higher than those in the control, OA_240, and ALA_240, respectively. The ratio of UFA to SFA can be used to as an indirect indicator of membrane fluidity [23]. The additions of OA, LA, and ALA increased membrane fluidity when compared to the control, as indicated by the increased ratio of UFA/SFA with enhancing concentrations of exogenous UFAs (Figure S2). Correspondingly, the highest ratio was observed in LA added wines, which might partially explain the higher cell growth rate and fermentation activity observed in these wines.

### 2.2. Volatile Compounds in the Wines

**Figure 2:**
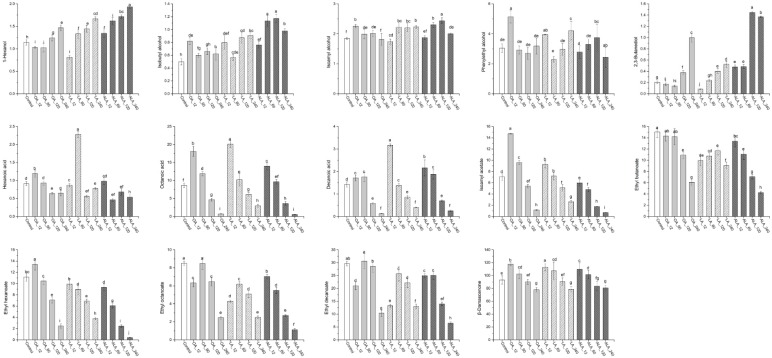
Odour active value (OAV) of main volatile compounds (OAV > 1) in wine with different treatments. Values of the same compound marked with the same lowercase letters indicates no significant difference (*p* < 0.05, Duncan’s test).

**Figure 3:**
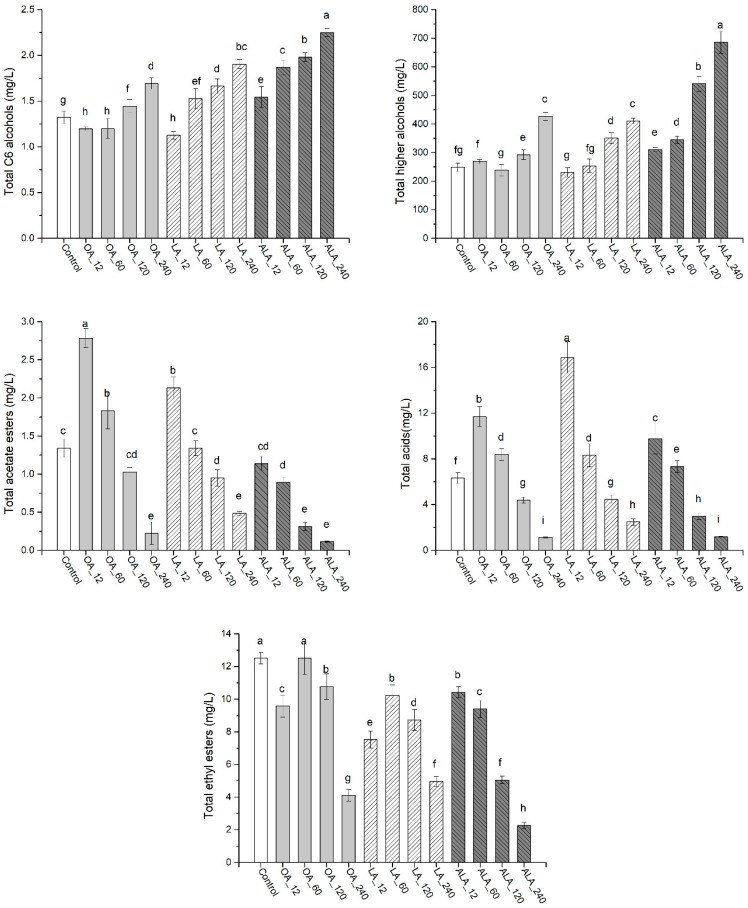
Total concentrations of C6 alcohols, higher alcohols, acetate esters, acids, ethyl esters, terpenes, and norisoprenoids in the final wines with different treatments. Values of the same compound marked with the same lowercase letters indicates no significant difference (*p* < 0.05, Duncan’s test).

A total of thirty-six yeast-derived volatile compounds were detected in all samples, including three C6 alcohols, seven higher alcohols, four medium-chain fatty acids, three acetate esters, eight ethyl esters, seven other esters, three terpenoids and norisoprenoids, and one methionol (Table S1). Thirteen compounds that odour active values (OAVs) exceed one and the total concentrations of each volatile group were presented in Figure 2 and Figure 3, respectively.

#### 2.2.1. C_6_ Alcohols and Higher Alcohols

C_6_ alcohols usually contribute “vegetal” and “herbaceous” note to wine, causing a negative effect on wine aroma profile. Three C_6_ alcohols, including 1-hexanol, (*E*)-3-hexen-1-ol and (*Z*)-3-hexen-1-ol were detected in this study, with only 1-hexanol exceeding threshold (Table S1, Figure 2). UFAs additions caused significant variation of C_6_ alcohol in wines, which were largely dependent on UFAs types and concentrations. Except for low concentrations of OA and LA (12 and 60 mg/L), other treatments resulted in increased contents of 1-hexanol and total C_6_ alcohols as compared to the control, especially ALA. For example, 1-hexanol in 240 mg/L ALA addition was 69.2%, 32.0%, and 15.9% higher than those of the control, OA_240 and AL_240, respectively, suggesting that ALA addition is more favorable for the biosynthesis of C_6_ alcohols when compared to other two UFAs. This could be ascribed to the fact that more C_6_ aldehydes and C6 alcohols can be generated from the degradation of ALA catalyzed by LOX/HPL pathway [24,25].

Higher alcohols are important yeast-derived volatile compounds in wine, and they are mainly derived from amino acids degradation via the Ehrlich pathway [5]. In this study, isoamyl alcohol (green and alcohol note) and 2-phenylethanol (rose and floral note) exceed their thresholds in all wines, isobutyl alcohol (sweet and alcohol note) was above the threshold only in ALA_60 and ALA_120 wines, while 2,3-butanediol (fruity, sweet and butter note) exceeded its threshold in OA_240, LA_240, ALA_120, and ALA_240 wines (Table S1, Figure 2). These data suggested that the formations of higher alcohols in response to exogenous UFAs were associated with compounds, UFAs type, and concentration. In general, increased UFAs content were beneficial for the formations of this group volatiles, which agreed with the previous literature results [15,19]. It should be emphasized that the promotion extents varied with UFAs types. ALA presented highest stimulation as the highest content were found in ALA_240 wine, which was 2.8-, 1.6-, and 1.7-fold higher than that in the control, OA_240, and LA_240, respectively (Figure 3). The maximum amount of 2,3-butanediol was observed in ALA_120, which was 6.1-, 7.5-, and 2.6-fold higher than those of the control, OA_120, and LA_120. Interestingly, the highest contents of 2-phenylethanol was found in OA_12 wine. Higher alcohol exceeding 400 mg/L can cause an unpleasant flavor to wine. The concentrations of higher alcohols in OA_240 (427 mg/L), LA_240 (411 mg/L), ALA_120 (541 mg/L), and ALA_240 (689 mg/L) exceed this value, suggesting that it is crucial to control UFAs concentration in must to reduce the contents of higher alcohols in final wine.

#### 2.2.2. Medium-Chain Fatty Acids

Fatty acids impart the wine with fruity, cheese, fatty, and rancid notes. Volatile fatty acids can improve the complexity of wine at sub-sensory threshold levels, but can cause negative effect on wine aroma when above their thresholds [8]. Four medium-chain fatty acids (MCFAs) were detected in this study. Hexanoic acid, octanoic acid, and decanoic acid exceeded their individual threshold (Table S1, Figure 2). Low concentrations of UFAs (12 and 60 mg/L) increased MCFAs productions, including octanoic acid, decanoic acid, and total contents when compared to the control, and their contents in LA_12 wine were 132.7%, 123.0%, and 167.2% higher than that of the control, respectively, and the values were also 43.7%, 46.8%, 72.7% higher than those of ALA_12, respectively. However, with the increment of UFAs concentrations (120 and 240 mg/L), this promotion disappeared and it conversely became the inhibition, regardless of UFA types. Exogenous UFAs (such as palmitoleic acid, linoleinic acid) can suppress the biosynthesis of fatty acids and MCFAs [9,21]. Our data showed that the inhibition only occurred at a high concentration of UFAs (above 120 mg/L in this study). Slight increment of UFAs content promoted MCFA formations. Among three UFAs, LA presented the highest promotion in the same concentration.

#### 2.2.3. Esters

Esters, including acetate esters and ethyl esters, are the primary source of fruity aromas and positively contribute to the desired fruit aroma characters of wine [7,26]. Three acetate esters were detected in this study, including isoamyl acetate, hexyl acetate, and phenethyl acetate, and only isoamyl acetate (banana note) exceeded its threshold (Table S1, Figure 2). Adding 12, 60 mg/L OA, and 12 mg/L LA increased the productions of acetate esters, and the highest total content was observed in OA_12 wine (107.6%, 30.5%, and 144.6%) higher than those of the control, LA_12 and ALA_12, respectively). Surprisingly, the promotion was not found in ALA added wine. Similar to MCFAs, increasing UFAs concentration deprived the stimulation on acetate esters and conversely became the inhibition. The highest inhibition was found in ALA_240 wine, followed by OA_240, (91.5% and 83.3% reduction as compared to the control, respectively). Exogenous UFAs can repress *ATF1* transcription to inhibit the biosynthesis of acetate esters [27]. Casu et al. found that adding 132 mg/L LA into Sauvignon Blanc reduced acetate esters formation, which was consistent with our results [14]. However, we found that the impact of UFAs on acetate esters is the concentration and type dependent. The low amount of OA and LA in must (below 60 mg/L in this work) showed the promotion (especially isoamyl acetate), with the exception of ALA, while high concentrations of UFAs (above 120 mg/L) caused the inhibitions, irrespective of UFA types.

Ethyl ester is another important group of esters in wine, which is produced through ethanolysis of acyl-CoA during yeast fermentation [7,8]. Eight ethyl esters were detected in all samples. Ethyl butanoate (banana, pineapple, and strawberry note), ethyl hexanoate (banana and green apple note), ethyl octanoate (sweet, floral, fruity, banana, and pear note), and ethyl decanoate (fruity and fatty note) exceeded their individual threshold (Table S1, Figure 2). Consistent with the results of previous literatures [14,22,26], except for ethyl hexanoate in OA_12 wine, adding UFAs reduced the formations of ethyl esters. Besides concentration, the extent of inhibition is varied with the type of UFAs. ALA had highest inhibition among three UFAs with the same levels of treatments. The lowest total content of ethyl esters observed in ALA_240 wine showed 81.9%, 45.1%, and 54.3% reduction when compared to the control, OA_240, and LA_240, respectively (Figure 3). The biosynthesis of ethyl esters is usually believed to be substrates (MCFAs) dependent [28]. In this study, high MCFAs (OA_12 and LA_12 wine) cannot correspondingly guarantee the higher production of ethyl esters, implying that regulations of aroma-related genes might be involved in ethyl esters formations, as stated by Saerens et al. [29].

#### 2.2.4. Norisoprenoids and Terpenes

Norisoprenoids and terpenes are derived from grapes and have relatively low thresholds. Thus, a small variation of their concentrations can result in significant influence on the entire aroma profiles of wines [8]. One norisoprenoid (β-damascenone) and two terpenes (citronellol and 4-terpineol) were detected in this study, with β-damascenone exceeding its threshold. (Table S1, Figure 2). The additions of 12 and 60 mg/L OA, LA, and ALA resulted in increased production of β-damascenone, but this promotion was weakened with increasing UFAs concentrations (Figure 3). The highest level of β-damascenone was found in LA_12 wine, followed by OA_12 and ALA_12 wine. There were no significant differences of β-damascenone in other treatments.

**Figure 4:**
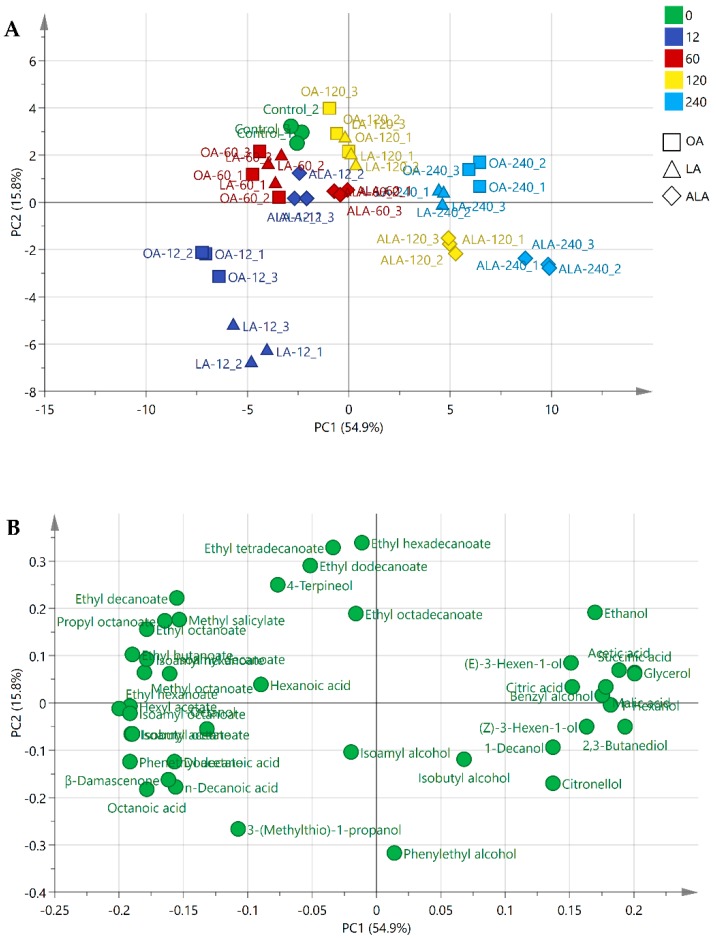
Principal component analysis (PCA) score plot (**A**) and loadings plot (**B**) using 36 volatile compounds and main fermentation products (including ethanol, glycerol, acetic acid, citric acid, malic acid, and succinic acid) in the Control (green circle), 12 mg/L (blue), 60 mg/L (red), 120 mg/L (yellow), and 240 mg/L mg/L (light blue) addition of oleic acid (OA, square), linoleic acid (LA, triangle), and α-linolenic acid (ALA, diamond).

The above results indicated that the production of volatile compounds in final wines was significantly affected by UFAs type and content in grape must, including C6 alcohols, higher alcohols, MCFAs, and esters. To highlight the differences in wines with different treatments, we carried out principle component analysis (PCA) using volatile compounds and main fermentation products (including ethanol, glycerol, acetic acid, citric acid, malic acid, and succinic acid) (Figure 4). As shown in Figure 4A, the first and second accounted for 54.9% (PC1) and 15.8% (PC2) of the total variation, respectively. The PCs clearly distinguished the wine samples that were fermented by the addition of OA, LA, with ALA. UFAs levels had stronger contribution to the variance of these compounds than UFAs types in OA and LA wines. OA_12 and LA_12 wines were located on the negative part of PC1 and they were clearly separated from other treatments. The main responsible components for this separation were MCFAs (octanoic acid and decanoic acid), acetate esters (hexyl acetate, phenethyl acetate, and isoamyl acetate), and ethyl esters (ethyl hexanoate, ethyl butanoate, and ethyl octanoate). In comparison, the OA_240 and LA_240 wines were positioned on the positive side of PC1 by higher alcohols (2,3-butanediol, hexanol, and benzyl alcohol), as well as ethanol, glycerol, succinic acid, and acetic acid. The PC2 could discriminate the wine of ALA from the wines of OA and LA, mainly by isobutyl alcohol, isoamyl alcohol, and 2,3-butanediol, suggesting that ALA addition could produce the wines having different enological properties with OA and LA wines.

### 2.3. Expression of Genes Related to Volatile Compounds Formation

**Figure 5:**
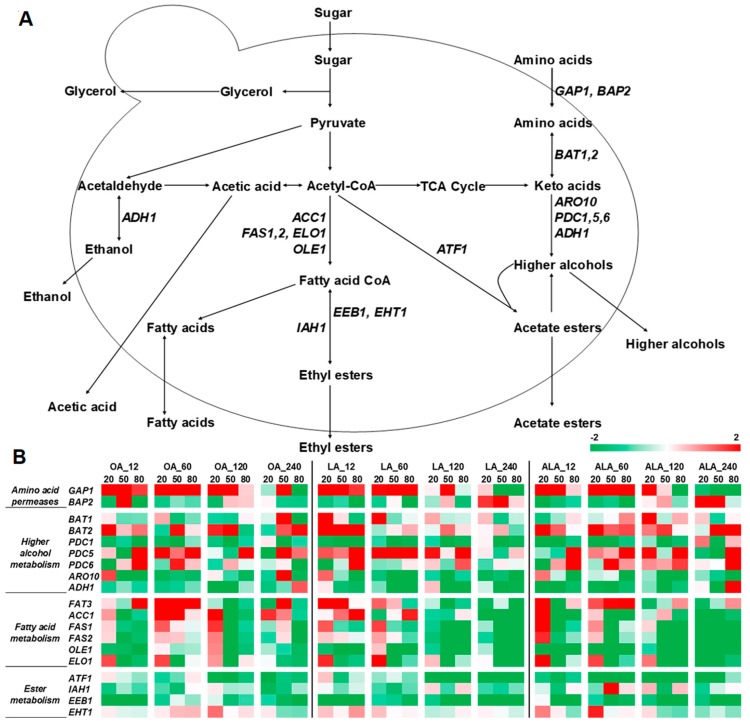
(**A**) Metabolic pathway involved in volatile compounds formation. Yeast genes that are involved in each step in the different pathways are shown in italic. (**B**) Comparative analysis of genes expressions of yeast in different treatments with that in the Control at each fermentation stage (sugar consumption 20%, 50%, and 80%) were conducted. The ratios were obtained using the corresponding Control as reference (red up-regulated and green down-regulated).

Transcriptome analyses of genes that are related to aroma production in *S. cerevisiae* have proven, to some extent, to be correlated with aroma compounds production during wine [21,30,31]. Given this, we have compared the relative expression levels of nineteen genes that are involved in the formation of different aroma compounds at three stages by Real-time PCR. The descriptions of these genes are listed in Table S2. The expressions of *S. cerevisiae* genes in UFAs added wines were compared with those of the control (Figure 5). The results show that, aside quantitative variation for each gene found within the different treatments, most of them displayed the similar trend in each fermentation. UFAs addition up-regulated most genes that are involved in amino acids transportation and metabolism during fermentation. The higher expression levels of genes involved in fatty acids metabolism were observed only in 12 and 60 mg/L UFAs addition samples, with the highest values in LA_12; while the genes related to esters formation were down-regulated in most of the treatments.

The formation of higher alcohols requires the activity of amino acids transporters, transaminases, decarboxylases, and dehydrogenases [30] (Figure 5). Amino acids permeases are mainly encoded by *GAP1* and *BAP2* genes, transaminases by *BAT1*, *BAT2* genes, decarboxylases by *PDC1*, *PDC5*, *PDC6,* and *ARO10*, and dehydrogenases by *ADH1* [5]. The increased contents of higher alcohols in OA, LA, and ALA wines (Figure 3) were in line with the higher expressions of most aroma genes, such as *GAP1*, *BAT1*, *BAT2*, *PDC5,* and *PDC6* comparing to the expression registered in the control. In addition, ALA_120 and ALA_240 induced higher expressions of *BAT1*, *BAT2*, *PDC1*, *PDC5,* and *PDC6* than those of other treatments (Figure 5). This could explain how higher levels of higher alcohols were generated in ALA wines. UFAs can modulate the activity of membrane-associated enzymes and transporters to increase higher alcohols production during wine fermentation (such as amino acids permeases GAP) [6,15,20]. Our data were corresponding to these results, and they further indicated that the up-regulations of transaminases (*BAT1* and *BAT2*) and decarboxylases genes (*PDC1*, *PDC5*, *PDC6,* and *ARO10*) were also involved in the enhancement of higher alcohols by UFAs addition.

Medium-chain fatty acids formation entails the activity of acetyl-CoA carboxylase and the fatty acid synthase (encoded by *ACC1*, *FAS1,* and *FAS2*). *ACC1* and *FAS1,* which are involved in the synthesis of medium-chain fatty acyl-CoA (MCFA-CoA), and their up-regulations, are usually accompanied with the high production of MCFAs [32]. *FAT3* encodes protein required for fatty acids uptake and *ELO1* involved in the elongation of fatty acids. Previous literatures demonstrated that exogenous UFAs could result in decreased production of FAs and UFAs in yeast by down-regulating *ACC1*, *FAS1,* and *FAS2*, and lead to low contents of MCFAs in wine [9,15,33]. In this study, we found that low concentrations of OA, LA, or ALA (12 and 60 mg/L) increased the expressions of *FAT3*, *ACC1*, *FAS1*, *FAS2,* and *ELO1*, and consequently resulted in high productions of MCFAs (Figure 3, Figure 5). It should be noticed that among these treatments, LA_12 and OA_60 additions presented higher expression profiles of *FAT3*, *ACC1*, *FAS1,* and *FAS2* during fermentation. However, MCFAs contents in both wines were largely different. LA_12 wine had the highest amounts of MCFAs (including total, octanoic acid, and decanoic acid), which were 101.4%, 69.1%, and 79.9% higher than those of OA_60 wine, respectively. This implied that there might be other functional genes involved in MCFAs formations under this condition. Strikingly, the expressions of all genes involved in fatty acid metabolism (*FAT1*, *ACC1*, *FAS1*, *FAS2*, *ELO1,* and *OLE1*) were significantly repressed by high UFAs addition (120 and 240 mg/L) during fermentation, especially LA and ALA samples. This explained the marked reduction of MCFAs contents in these wines relative to the control. *OLE1* encodes delta (9) fatty acid desaturase and it is responsible for catalyzing FAs form UFAs. Exogenous UFAs expressed the expression of *OLE1* significantly because cells do not need de novo synthesis of UFAs from MCFAs and FAs [33]. Similar results were found in the present study.

*ATF1* encodes the alcohol acetyl transferase (AATases) and it is involved in the biosynthesis of acetate ester [7,34]. Its expression is positive with the production of acetate esters in wine. Exogenous UFAs can repress the expression of *ATF1* and results in decrement of acetate esters in wine [35]. Similar phenomena were observed in 120 and 240 mg/L OA and LA addition wines (OA_120, OA_240, LA_120, and LA_240), as well as all ALA addition wines (Figure 5). However, no continuous and significant repressive effects on *ATF1* expression were observed in OA_12, OA_60, and LA_12 fermentation, conversely, slight increased expressions were found in the early phase of OA_12 and LA_12 fermentation, and the late phase of OA_60 fermentation relative to the control, respectively, which might, at least partially, account for increased productions of acetate esters in these wines (Figure 3, Figure 5). At present, we cannot exclusively ascribe these increments to the up-regulation of ATF1, because increased number of cells could also result in the accumulation of acetate esters [15]. Exogenous UFAs can result in the inhibition of biosynthesis of acetate esters [12,14]. Our data indicated that the inhibition is mainly occurred in the higher concentrations of OA and LA and ALA added fermentations (Figure 3).

In contrast to acetate ester production, the formation of MCFA ethyl ester is more complicated. The rate of MCFA ethyl ester formation is dependent on the concentration of the two substrates (the acyl-CoA component and ethanol) and the total activity of the enzymes that are involved in the synthesis (encoded by *EEB1* and *EHT1*) and hydrolysis of the MCFA ethyl esters (encoded by *EHT1*). Saerens et al. found that the maximum expression level of *EEB1* is correlated with ethyl hexanoate concentration, but not ethyl octanoate and decanoate [29]. Due to Eht1 possessing both synthesis and hydrolysis activity towards ethyl esters, there is a strong negative correlation between *EHT1* expression levels and the end concentrations of ethyl octanoate and decanoate [29]. In this study, we found that *EEB1* was repressed in UFAs added fermentation, and *EHT1* were simultaneously up-regulated, which might explain the pronounced reduction of ethyl ester in UFAs addition fermentation as compared to the control (Figure 5). Some authors believed that precursor concentrations (MCFA) are the limiting factor for ethyl ester synthesis, because the addition of MCFA to the fermentation medium resulted in higher ethyl ester production [28]. We did not observe the corresponding relationship between MCFAs and ethyl esters in OA_12 and LA_12 wine, although high MCFAs were produced in both wines (Figure 3). Conversely, there was a great reduction of ethyl esters contents, which was in line with the down-regulation of *EEB1* and up-regulation of *EHT1* (Figure 5). Our data indicated that the formation of ethyl esters was not only associated with substrate levels, but also the regulations of ester synthesis genes.

## 3. Materials and Methods

### 3.1. Yeast Strain and Culture Medium

The commercial wine yeast *S. cerevisiae* var. *bayanus* strain EC1118 (Lallemand, Blagnac, France) was used in this study. For the fast and robust fermenting profile and the neutral contribution to the wine aroma, this strain is used worldwide for both red and white winemaking [36]. *Vitis vinifera* Cabernet Sauvignon must, which was prepared commercially from grapes harvested from Changli wine region of China in 2017, was used for wine fermentation. 60 mg/L sulfur dioxide was added into the must after the de-stemming and crushing, and 48 h of maceration at 8 °C was performed before the skin and seeds were removed. The initial sugar concentration was 241.3 g/L, and OA, LA, and ALA in must were 1.24 mg/L, 2.02 mg/L, and 0.55 mg/L, respectively. Four levels (12 mg/L, 60 mg/L, 120 mg/L and 240 mg/L) of additional OA, LA, and ALA were designed according to our preliminary work [37] and introduced in initial grape juice, which were set as OA_12, OA_60, OA_120, OA_240, LA_12, LA_60, LA_120, LA_240, ALA_12, ALA_60, ALA_120, and ALA_240, respectively. The juice without UFAs addition was set as the control. The final concentration of UFAs was within the common range in grape must [15,16].

### 3.2. Fermentation Conditions and Sampling

After being inoculated into 500 mL yeast extract peptone dextrose (YPD) medium (5 g/L yeast extract, 10 g/L peptone, and 20 g/L glucose), *S. cerevisiae* was incubated at 30 °C with shaking (150 rpm) overnight. Yeast cells were harvested and washed twice with sterile water before being inoculated into grape must. The initial density of cells was 10^6^ CFU/mL. Fermentations were carried out in triplicate in 500 mL flasks that were equipped with fermentation locks and sampling needles and contained 350 mL of must at 25 °C without shaking.

Cell density (optical density at 600 nm, OD_600 nm_) and sugar consumption was used to monitor the fermentation progress. Dry cell weight (DCW) was obtained by DCW (g/L) = 0.3 × OD 600 nm based on the methods of Molina et al. [36]. 30 mL samples of culture medium were taken from initial musts and fermenting musts in mid-exponential (20% sugar consumption), early-stationary (50% sugar consumption), late-stationary (80% sugar consumption) growth phase and final wine, and centrifuged for 10 min at 13,800× *g*. The cells were harvested and kept at −80 °C for RNA isolation, while supernatants were kept at −20 °C for the detection of main fermentation products, fatty acids, and volatile compounds.

### 3.3. Analysis of Basic Wine Compounds

Glycerol, ethanol, and organic acids (including acetic acid, citric acid, succinic acid, and malic acid) in the samples were measured by high-performance liquid chromatography 1200 series (HPLC, Agilent Technologies, Santa Clara, CA, USA) using an HPX-87H Aminex ion-exchange column (300 mm × 7.8 mm, Bio-Rad Laboratories, Hercules, CA, USA), as described previously [37]. A flow rate of 0.6 mL/min was used with 5 mmol sulfuric acid as the mobile phase. After being filtered through a 0.22 μm membrane filter (Dikma Technologies, Lake Forest, CA, USA) before HPLC analysis, 2 μL of the samples were injected. Glycerol and ethanol were detected by a refractive index detector (Agilent Technologies, Santa Clara, CA, USA) with the column maintained at 45 °C. Acetic acid, citric acid, succinic acid, and malic acid were detected by a photodiode array detector (Agilent Technologies, Santa Clara, CA, USA) at 214 nm with the column at 60 °C. Analyses were carried out in triplicate. The method quantitation limit (MQL), calibration curves, and R^2^ of glycerol, ethanol, acetic acid, citric acid, succinic acid, and malic acid used in this study are provided in Table S3.

### 3.4. Analysis of Fatty Acids and Volatile Compounds

Fatty acids in the yeast cells were analysed according to the method of Duan et al. [15]. 5–10 mg dry weight yeast cells were placed in sealed tubes then saponified at 100 °C for 30 min with 1 mL of 5% NaOH in 50% methanol/water. 2 mL HCl (6 M) was added to the tubes. Free fatty acids were extracted with 0.5 mL hexane: methyl-tert-butyl ether (1:1, *v*/*v*). The gas chromatograph-mass spectrum (GC-MS) system used was a 6890A gas chromatograph equipped with a 5975C mass spectrum system (GC-MS, Agilent Technologies, Santa Clara, CA, USA). The column used was a 60 m × 0.25 mm × 0.25 μm HP-INNOWAX column (J&W Scientific, Folsom, CA, USA) under 1 mL/min helium carrier gas. Oven starting temperature was 80 °C (held for 1 min), then raised to 220 °C at a rate of 25 °C /min, and then from 220 °C to 250 °C (held for 20 min) at 5 °C/min. The mass spectrometer (Agilent Technologies, Santa Clara, CA, USA) in the electron impact (EI) mode at 70 eV was recorded in the range *m/z* 20 to 350. Analyses were carried out in triplicate. The detailed quantitation information about quantitative ion, quantitative standards, MQL, calibration curves, and R^2^ for the quantification of fatty acids used in this study is provided in Table S4.

The extraction and analysis of wine volatile compounds were based on the available methods developed by our laboratory [38,39]. After extraction with SPME fiber (50/30 μm DVB/CAR/PDMS, Supelco, Bellefonte, PA, USA), samples (5 mL of wines) were analysed by an Agilent 6890A and a column HP-INNOWAX (60 m × 0.25 mm × 0.25 μm) equipped with a 5975C MS system. The qualification and quantification process were based on our lab previous work [39]. The detailed quantitation information about quantitative ion, quantitative standards, MQL, calibration curves, and R^2^ for the quantification of volatile compounds used in this study is provided in Table S5.

### 3.5. RNA Extraction and Real-Time qPCR Assay

RNA of yeasts in mid-exponential (20% sugar consumption), early-stationary (50% sugar consumption), and late-stationary (80% sugar consumption) growth phase cells of strain (Table S6) was extracted with hot phenol method as described by Deed et al. [4]. cDNA was synthesized and real-time PCR was performed according to our previous method [37]. The descriptions and primers of the selected genes were listed in Table S2. *PDA1* and *ACT1* were taken as internal controls according to the previous research [40]. The normalized expression of target genes was obtained according to Livak & Schmittgen [41]. Three independent extraction procedures were applied for each sample and two technical replications of Real-time PCR analysis were performed.

### 3.6. Statistical Analysis

One-way analysis of variance (ANOVA) to test for significant difference in metabolites among treatments was assessed by the Duncan test (*p* < 0.05) with SPSS20.0 software (SPSS, Chicago, IL, USA). Principle component analysis (PCA) was performed by SIMCA 14.1 (Umetrics, Malmö, Sweden). The rest graphs were performed by Origin 9.0 (OriginLab Corporation, Northampton, MA, USA).

## 4. Conclusions

The results of this study indicated that UFAs type and content in grape must significantly influence cell growth, the fermentation process, and the productions of yeast-derived volatile compounds. Initial adding low concentrations of UFAs (12 and 60 mg/L), especially LA and OA, promoted cell growth, fermentation activity, and most volatiles when compared to the control. However, this effect disappeared and conversely became the inhibition with the increment of UFAs concentrations (120 and 240 mg/L). Regarding the individual aromatic effects of different UFAs, low concentration of OA and LA (12 and 60 mg/L) enhanced the productions of MCFAs (octanoic acid and decanoic acid) and acetate esters (isoamyl acetate), in which OA had a pronounced effect on acetate esters biosynthesis, while LA facilitated more MCFAs formation. In comparison, ALA with low concentration had no effect on acetate esters production. Conversely, the supplementation of 120 and 240 mg/L ALA greatly promoted the productions of C6 alcohols (1-hexanol) and higher alcohols (isobutyl alcohol and 2,3-butanediol). The additions of UFAs were unfavorable for the biosynthesis of ethyl esters, with the exception of ethyl hexanoate in 12 mg/L OA added fermentation. As a result, different aromatic profiles of wines were produced by the variation of UFAs types and levels showed by PCA. The enological characteristics of ALA wines were distinct with OA and LA wines. UFAs levels had a stronger contribution to the variance of the aromatic compounds than UFAs type in OA and LA wines. The transcriptional data revealed that the expressions of aroma-related genes, such as *BAT1*, *BAT2*, *PDC1*, *PDC5*, *PDC6*, *ACC1*, *FAS1*, *ATF1*, *EEB1,* and *EHT1* were correlated with aroma compounds production in different fermentations. Our results collectively suggested that different UFAs have no similar enological effects during red wine fermentation. Thus, besides concentration, it is essential to consider the types of UFAs in grape must when applying the strategy of adjusting UFAs contents to modulate the aromatic quality of wine products, which might be a potential way to increase the aromatic diversity and quality of wine products. For example, to obtain a wine with high fruity or floral characters, the regulation of grape must to contain low content of OA or LA could be taken into consideration. On the other hand, if a wine is required to have relative high alcohols for further storage, a grape must with high ALA could be a good choice. Future work will be done to further confirm the individual aromatic effect of different UFAs in other grape varieties, such as Melort and Sauvignon Blanc being fermented by different *S. cerevisiae* strains.

## Figures and Tables

**Table 1:** Effect of different treatments on final fermentation values.

	Time to Reach the End of Fermentation (h)	Maximum Biomass (g DCW·L^−1^)	Time to Reach Maximum Biomass (h)	Maximum Specific Growth Rate (h^−1^)	Maximum Fermentation Rate (g·L^−1^·h^−1^)
Control	250	2.15 ± 0.08 ^g^	84	0.008 ± 0.000 ^g^	2.77
OA_12	108	2.96 ± 0.11 ^ab^	84	0.022 ± 0.001 ^b^	5.02
OA_60	132	2.80 ± 0.24 ^bc^	84	0.015 ± 0.000 ^cd^	3.24
OA_120	250	2.78 ± 0.10 ^bc^	72	0.009 ± 0.001 ^f^	3.73
OA_240	444	2.34 ± 0.33 ^fg^	96	0.005 ± 0.000 ^h^	2.11
LA_12	96	3.08 ± 0.11 ^ab^	60	0.023 ± 0.002 ^a^	7.97
LA_60	132	3.00 ± 0.15 ^ab^	72	0.016 ± 0.001 ^c^	3.58
LA_120	250	2.85 ± 0.14 ^abc^	72	0.01 ± 0.001 ^f^	3.49
LA_240	444	2.53 ± 0.13 ^def^	108	0.005 ± 0.000 ^h^	2.6
ALA_12	132	2.61 ± 0.18 ^cde^	84	0.015 ± 0.000 ^d^	3.71
ALA_60	180	2.84 ± 0.05 ^abc^	84	0.014 ± 0.001 ^e^	3.35
ALA_120	444	2.78 ± 0.14 ^bcd^	84	0.005 ± 0.000 ^h^	3.03
ALA_240	444	2.39 ± 0.09 ^ef^	84	0.005 ± 0.000 ^h^	1.48

Values (mean SD, *n* = 3) of the same parameter followed with the same lowercase letters indicated no significant difference (*p* < 0.05, Duncan’s test). DCW, dry cell weight.

## Supplementary Materials

The following are available online. Figure S1: Fermentation profiles of different treatments. Progress of biomass and sugar concentration throughout the fermentation. Biomass is reported as dry cell weight (DCW). Data points represent the mean values from triplicate fermentations and the vertical bars show ± SD. Figure S2: Effect of different treatments on the composition of fatty acids (SFAs, saturated fatty acids; UFAs, unsaturated fatty acids) in yeast cells. Values of the same compound marked with the same lowercase letters indicates no significant difference (*p* < 0.05, Duncan’s test). Table S1: Effect of UFAs on the concentration (μg/L) of volatile compounds in wines with different treatments. Table S2: Description of genes detected in this work. Table S3: Calibration curves for quantification of glycerol, ethanol, citric acid, malic acid, succinic acid, lactic acid and acetic acid in this study. Table S4: Quantitative ion, quantitative standards and calibration curves for quantification of fatty acids in this study. Table S5: Quantitative ion, quantitative standards and calibration curves for quantification of volatile compounds in this study. Table S6: Time of sampling of the alcoholic fermentation for Real-time PCR.
