# Supplementary material for: Comparing the Effects of Different Unsaturated Fatty Acids on Fermentation Performance of Saccharomyces cerevisiae and Aroma Compounds during Red Wine Fermentation
Source: Molecules. 2019 Feb 1;24(3):538. doi: 10.3390/molecules24030538 (PMC6384975; doi:10.3390/molecules24030538)
Supplement: Supplementary file 1 [file molecules-24-00538-s001.zip › Supplementary Figures-0110.pdf]

Supplementary Figures

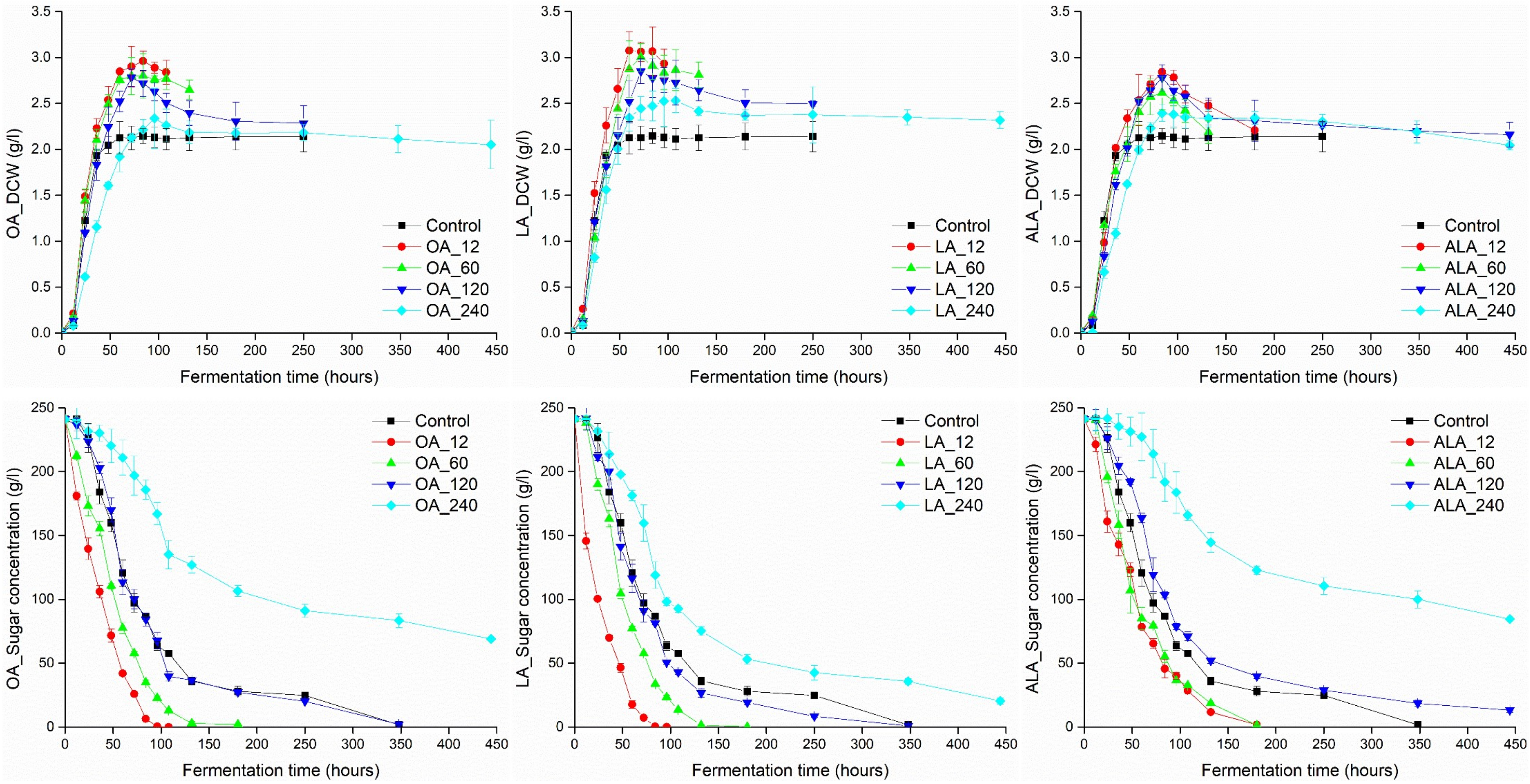

Figure S1. Fermentation profiles of different treatments. Progress of biomass and sugar concentration throughout the fermentation. Biomass is reported as dry cell weight (DCW). Data points represent the mean values from triplicate fermentations and the vertical bars show ± SD.

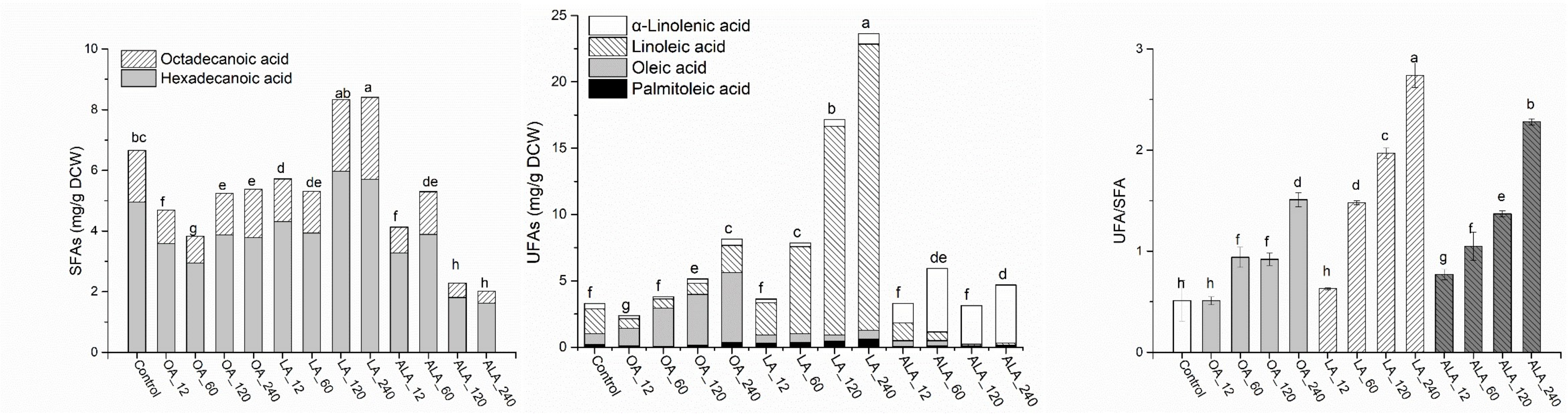

Figure S2. Effect of different treatments on the composition of fatty acids (SFAs, saturated fatty acids; UFAs, unsaturated fatty acids) in yeast cells. Values of the same compound marked with the same lowercase letters indicates no significant difference (*P*< 0.05, Duncan's test).
